# Parent-reported Mental Health Problems and Mental Health Services Use in South Australian School-aged Children

**DOI:** 10.3934/publichealth.2016.4.750

**Published:** 2016-09-22

**Authors:** Jing Wu, Eleonora Dal Grande, Helen Winefield, Danny Broderick, Rhiannon Pilkington, Tiffany K Gill, Anne W Taylor

**Affiliations:** 1 Population Research and Outcome Studies (PROS), School of Medicine, University of Adelaide, PO Box 498, Adelaide, South Australia 5001; 2 School of Psychology, University of Adelaide, South Australia 5005; 3 Public Health, Torrens University, South Australia 5000; 4 School of Public Health, University of Adelaide, South Australia 5005

**Keywords:** Children and adolescent, mental health problems, mental health services utilisation

## Abstract

**Background:**

Monitoring and reporting childhood mental health problems and mental health services utilization over time provide important information to identify mental health related issues and to guide early intervention. This paper aims to describe the recent prevalence of parent-reported mental health problems among South Australian (SA) children; to identify mental health problems associated characteristics; and to describe mental health services utilization and its related characteristics among this population.

**Methods:**

Parent-reported mental health problems were assessed against the first item of the Strength and Difficulties Questionnaire. School-aged children were randomly sampled monthly and data were collected using a surveillance system between 2005 and 2015. Associations between mental health problems and various factors were analysed using univariable analysis and multivariable logistic regression modelling.

**Results:**

Prevalence of parent-reported mental health problems among children was 9.1% and 9.3% for children aged 5 to 11 years and children aged 12 to 15 years, respectively. No change in prevalence was observed during the past decade. Mental health problems were associated with male sex, long-term illness or pain, negative school experiences, not living with biological parents, and living in a rental dwelling. Less than half (48.7%) of the children with mental health problems received professional help. An increasing trend was found in mental health services utilisation among children aged 5 to 15 years. Utilization of mental health services was associated with male sex, older age, long-term illness or pain, and feeling unhappy at school.

**Conclusion:**

This study reports the prevalence of parent-reported mental and mental health services utilisation among SA school-aged children. Identified characteristics associated with mental health problems and mental health services utilisation provide useful information for the planning of catered population initiatives.

## 1. Introduction

Children with mental health problems, either diagnosable disorders or milder temporary emotional and behavioural difficulties, have an increased risk of: reduced health and well-being [1], impaired quality of life [2], a greater likelihood of developing mental illness into adulthood [3], and compromised education and employment attainment [4]. Childhood mental health problems have significant adverse impacts on individuals, families and communities [5]. Mental health conditions are the leading cause of disability for children younger than 15 years in Australia and the major contributor to the substantial rise in childhood disability over the last decade [6].

Previous studies have reported that the prevalence of child mental health problems ranges between 10% and 20% globally [7]. A recent Australian survey showed that 14% children and adolescents aged 4 to 17 years had diagnosable mental health problems in the previous 12 months [8]. Limited information exists regarding the change in prevalence of mental health problems in school-aged children and adolescence over time because of the widespread use of single-phase, cross-sectional surveys with relatively long time gaps before they are repeated. It would be useful to report childhood mental health problem prevalence estimates in a timely manner and their variation over time. Timely and quality data enable public health agencies and service providers to monitor community health issues and provide feedback for preventive intervention programs to help increase their effectiveness in delivering clinical services [9].

About half of all mental health problems start before the age of 14 years [10]. Early recognition and effective treatment of mental health disorders can result in better outcomes [11]. The Strength and Difficulties Questionnaire (SDQ) has been used routinely as a screening tool to establish caseness for receiving specialised child and adolescent mental health services in Australia [8]. High scores indicate there is a considerable risk of clinically significant problem. Mental health problems measured using the first item of SDQ may include disorders of psychological development, behavioural and emotional disorders, as well as temporary difficulties at developmental phases. Although this may not correspond to a definite diagnosis or be stable over time, information is useful in identifying children who may need special attention. Sub-threshold children are most at risk of “slipping through the gap” and not receiving adequate help, as the proportion of children who receive specialised treatment is low [7],[12]. It remains an imperative goal to monitor treatment-seeking behaviour and identify vulnerable children in order to refine treatment and support services and develop preventative strategies.

Several common characteristics or risk factors for childhood and adolescence mental health problems have been documented. Children with poorer physical health are more likely to have mental health complaints [13]. Family circumstances and parenting style are important environmental factors that influence a child's behaviour and mental health. Earlier studies have indicated that family poverty or hardship, step-/blended families and single-parent household, low level of parental education, maternal psychological distress, both abusive and protective parenting practices are associated with increased mental health problems in children and adolescents [12],[14]–[18]. As children spend increasing amount of time with peers during middle childhood and adolescence, social incompetence and adverse school experiences may also contribute to childhood mental health problems [19].

This paper aims to use representative surveillance data to: (1) describe the prevalence of parent-reported emotional, social or behavioural problems among South Australian (SA) children; (2) identify the associated demographic, school experiences and general health characteristics; (3) describe the mental health service utilisation by this group; and (4) identify the help-seeking behaviour and associated demographic, school experiences and general health characteristics in this population.

## 2. Data and Methods

The SA Monitoring and Surveillance System (SAMSS) is a telephone survey system designed to monitor chronic diseases, risk factors and other health-related issues on a regular and ongoing basis [20]. Since 2002, a random cross-sectional sample of 600 people of all ages is interviewed monthly, selected from all households in SA with a connected telephone number listed in the Electronic White Pages (EWP). A letter of introduction is sent to the selected household and the person who was last to have a birthday within a 12-month period is chosen for interview. Interviews are conducted by a trained interviewer via a Computer Assisted Telephone Interview (CATI) system. Surrogate interviews are undertaken for persons in the household under the age of 16 by the most appropriate person (determined by the person who answered the telephone call) to answer on their behalf. Up to ten call backs are made in an attempt to interview the selected person; there are no replacements for non-respondents. Ethics approvals were obtained from the Human Research Ethics Committee of The University of Adelaide and the SA Department of Health. All participants gave informed verbal consent to participate in the telephone interview.

This study used data collected between July 2005 and June 2015, including 3973 children aged 5 to 11 years and 2643 children aged 12 to 15 years. The majority of surrogate interviews were conducted by the child's mother or step-mother (76.5%), followed by the child's father or step-father (21.1%) and other relative (2.4%). For ease of interpretation, ‘parent’ is used throughout this manuscript rather than specific parental/carer relationship. The average monthly response rate over ten years was 63.4% ranging from 51.4% to 75.2% with a decreasing trend over the past ten years.

*Mental health problems.* Problems with mental health were assessed using a single question “Overall, does (child) have trouble with emotions, concentration, behaviour or getting on with people?” which is based on the first item of the impact supplement of the Strengths and Difficulties Questionnaire (SDQ) [21]. Those who reported “quite a lot” or “very much” were categorized as having a mental health problem.

*Mental health services use.* Perceived need for treatment was assessed by asking respondents whether they thought their child needed “special help” for their mental health problems. Information regarding service utilisation was obtained through responses to: “Has (child) ever been treated for an emotional, mental health or behavioural problem?” and “Who has treated (child)?”

*Family demographic and socioeconomic characteristics.* Variables included age, gender, gross annual household income, socio-economic disadvantage of neighbourhood at an environmental level (using postcode classified into the Socio-Economic Index For Areas (SEIFA) 2011 Index of Relative Socio-Economic Disadvantage Quintiles [22], family structure, home ownership status, and the highest education of parents.

*School experience and health status.* Respondents were asked about the child's experiences at school in the previous month, including happiness at school, friends, and bullying. Health status was defined by whether the child had a long term illness or pain that puts strain on family. These questions were adapted from previous state-wide surveys in South Australia and Western Australia.

*Statistical analysis.* Data were analysed using Statistical Package for the Social Sciences for Windows version 22.0 [23] and STATA version 13.0 [24]. To reduce potential biases and to adjust for non-response, data were weighted by age, gender, area and probability of selection in the household to the most recently estimated resident population data when they became available (the 2001, 2006 and 2011 Australian census data). Main reasons for non-participating the survey are refusal and non-contacts.

The prevalence is presented as a fractional polynomial plot with time in quarters as the continuous variable. Chi-square tests were used to compare differences between categories. Logistic regression, including a time and age interaction term, was used for trend analyses of prevalence over time. Univariable analyses were conducted to identify any associations between a number of selected socio-demographic, school experience and health status factors and childhood mental health problem. Variables with a *p* ≤ 0.25 at the univariable level [25] were included in multivariable logistic regression models to investigate their associations with childhood mental health. All models were checked for collinearity of variables and no collinearity was evident for any of the models. Final regression models were obtained using backward stepwise elimination of non-significant variables based on the log likelihood ratio tests. Children at different stages of life (5–11 years and 12–15 years) were analysed separately. A *p* < 0.05 was regarded as statistically significant.

## 3. Results

**Figure 1.**
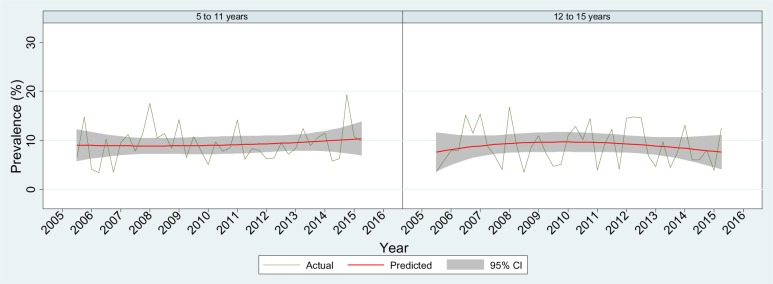
Prevalence of parent-reported mental health problems among children by age group 2005/06–2014/15.

**Figure 2.**
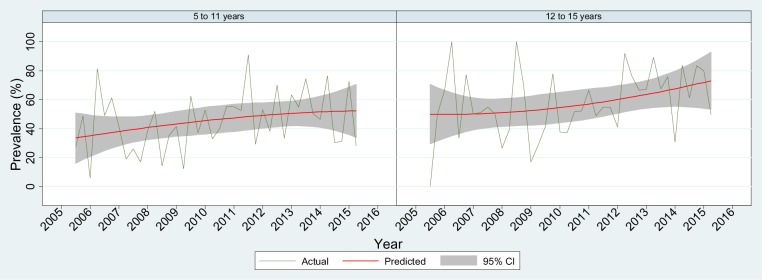
Prevalence of parent-reported mental health service use among children with mental health problems by age group, 2005/06–2014/15.

Table 1 describes the demographic characteristics of the study population. Overall, the prevalence (Table 1) of parent-reported emotional, behavioural or social trouble among children aged 5 to 11 years and children aged 12 to 15 years were similar (9.1% and 9.3%, respectively). In both age groups, the prevalence of mental health problems was twice as high for boys than for girls (12.3% vs 5.6% and 12.5% vs 5.8%, respectively). Over two-thirds of the parents (Table 1) who reported their child had mental health problems thought specific help from professionals was needed; however, about half of these children had actually been treated (44.8% for children aged 5 to 11 years and 54.4% for children aged 12 to 15 years, respectively). During the study period, the prevalence of parent-reported mental health problems remained unchanged over time for both age groups (Figure 1). An overall increasing trend in mental health service usage was observed among school-aged children (*p* for trend = 0.019). However, when analysed by age group (Figure 2), this statistically increasing trend was not found in either age groups. Of those who reportedly had mental health problems and received treatment, psychologist, school counsellor and paediatrician were the most popular choices (Table 2).

**Table 1.** Characteristics and prevalence of parent-reported mental health problems and seeking treatment in children by age group, 2005/06 to 2014/15.

	5 to 11 years (n = 3973)	12 to 15 years (n = 2643)
Age (mean ± SD)	8.1 ± 2.0	13.6 ± 1.1
Sex (male %)	51.0	50.4
Parent-reported mental health problems (Yes% (CI))	9.1 (8.2–10.0)	9.3 (8.2–10.4)
Female	5.6 (4.7–6.8)	5.8 (5.1–6.5)
Male	12.3 (11.0–13.8)	12.5 (11.6–13.5)
Perceive treatment needed among those with mental health problems (Yes% (CI))	67.6 (62.6–72.2)	74.9 (69.1–79.9)
Received treatment among those with mental health problems (Yes% (CI))	44.8 (39.7–50.0)	54.4 (48.1–60.6)

SD: standard deviation; CI: confidence interval.

**Table 2.** Mental health service used by children who had parent-reported mental health problems by age group, 2005/06 to 2014/15.

	5 to 11 years		12 to 15 years	
	n	%	n	%
Psychologist	81	50.5	71	53.6
School counsellor	37	23.3	45	33.8
Paediatrician	51	32.0	33	25.2
Psychiatrist	27	16.8	33	25.0
General practitioner	19	11.7	28	21.2
Complementary and alternative medicine practitioner	20	12.7	18	13.6
Social worker	8	5.3	19	14.0
Youth worker	6	3.5	10	7.5
Neurologist	5	3.3	5	3.8
Child and Youth Health	5	2.9	4	2.7
Other/Don't know	49	30.9	24	17.9

Multiple responses are possible; therefore, total service use is not shown.

The results of univariable analyses exploring the association between parent-reported mental health problems and socio-demographic attributes, general health- or school-related risk factors for the two age groups are presented in Table 3 and Table 4. Risk factors are similar for children and adolescents in the present study. Multivariable regression analyses showed that boys (OR 5–11 yrs = 2.37, 95% CI [1.83–3.08] and OR 12–15 yrs = 2.95, 95% CI [2.12–4.11]), not living with biological parents (OR 5–11 yrs = 1.52, 95% CI [1.13–2.05] and OR 12–15 yrs = 1.65, 95% CI [1.17–2.33]), having long-term illness or pain (OR 5–11 yrs = 5.04, 95% CI [3.80–6.70] and OR 12–15 yrs = 4.17, 95% CI [2.84–6.10]), and adverse school experiences, such as being unhappy at school (OR 5–11 yrs = 3.45, 95% CI [2.63–4.54] and OR 12–15 yrs = 5.04, 95% CI [3.58–7.05]), not having a group of friends or best mate to play with (OR 5–11 yrs = 3.54, 95% CI [2.47–5.06] and OR 12–15 yrs= 5.74, 95% CI [3.59–9.18]) and being bullied in last month (OR 5–11 yrs = 1.58, 95% CI [1.20–2.09] and OR 12–15 yrs = 1.91, 95% CI [1.34–2.72]) were associated with increased risk of mental health problems among children in both age groups (Table 5). Currently living in a rental dwelling was associated with increased risk of mental health problem among the children age 5–11 years (OR = 1.43, 95% CI [1.03–1.99]) but not the children aged 12–15 years.

Table 6 shows the results of univariable analysis exploring the association between treatment-seeking behaviour and socio-demographic characteristics, health and school-related risk factors for all children who had parent-reported mental health problems. Multivariable regression analyses (Table 7) indicate that older children (OR = 1.55, 95% CI [1.09–2.22]), being male (OR = 1.48, 95% CI [1.01–2.17]), having long-term illness or pain (OR = 4.10, 95% CI [2.82–5.94]), as well as those who often felt unhappy at school (OR = 2.35, 95% CI [1.59–3.49]), were more likely to seek professional help for their mental health problems.

**Table 3.** Univariable analysis of association between demographic, risk factors and parent-reported mental health problems among children aged 5 to 11, 2005/06 to 2014/15 (n = 356/3973).

	n/N	%	Odds Ratio	95% Confident Interval	*P* value
**Sex**					
Female	109/1940	5.6	1.00		
Male	249/2024	12.3	2.35	1.86–2.97	**< 0.001**
**Household income ^1^**					
≥$40,001	223/2985	7.5	1.00		
≤$40,000	84/557	15.0	2.19	1.67–2.87	**< 0.001**
**SEIFA quintile**					
Middle/High/highest quintile	190/2494	7.6	1.00		
Low/lowest quintile	166/1464	11.3	1.55	1.25–1.93	**< 0.001**
**Family structure ^1,2^**					
Living with biological parents	248/3247	7.6	1.00		
Step or blended family/sole parent family/shared care/other	104/687	15.1	2.15	1.69–2.75	**< 0.001**
**Current dwelling ^1^**					
Owned/being purchased	279/3407	8.2	1.00		
Rented from government/rented privately/other	80/549	14.6	1.91	1.46–2.50	**< 0.001**
**Highest education of parents ^1^**					
University degree or higher	83/1231	6.7	1.00		
Trade/certificate/diploma or lower	276/2730	10.1	1.55	1.20–2.00	**0.001**
**Long term illness or ongoing pain that puts a strain on family ^1^**					
No	225/3581	6.3	1.00		
Yes	134/379	35.3	8.12	6.32–10.43	**< 0.001**
**Ever unhappy at school ^1^**					
Never/Rarely	116/2721	4.3	1.00		
Sometimes/Often/Always	232/1121	20.7	5.84	4.62–7.40	**< 0.001**
**Have a best mate/a group of friends ^1^**					
Yes	264/3635	7.3	1.00		
No	80/212	37.8	7.75	5.71–10.51	**< 0.001**
**Bullied in last month ^1^**					
No	173/2864	6.0	1.00		
Yes (physical/emotional/both)	169/929	18.2	3.46	2.76–4.34	**< 0.001**

^1^ Other/not stated category not reported. ^2^ The “blended family” category within family structure was defined to include grandparents caring for grandchildren and foster care arrangements. Abbreviations: n, number of children with parent-reported mental health problems; N, total number of children in the relevant category; SEIFA, Socio-Economic Index For Areas.

**Table 4.** Univariable analysis of association between demographic, risk factors and parent-reported mental health problems among children aged 12 to 15, 2005/06 to 2014/15 (n = 244/2643).

	n/N	%	Odds Ratio	95% Confident Interval	*P* value
**Sex**					
Female	77/1308	5.9	1.00		
Male	167/1330	12.5	2.27	1.72–3.01	**< 0.001**
**Household income ^1^**					
≥ $40,001	156/1943	8.0	1.00		
≤ $40,000	64/388	16.4	2.25	1.64–3.08	**< 0.001**
**SEIFA quintile ^1^**					
Middle/High/highest quintile	134/1676	8.0	1.00		
Low/lowest quintile	110/953	11.5	1.49	1.14–1.95	**0.003**
**Family structure ^1,2^**					
Living with biological parents	143/2008	7.1	1.00		
Step or blended family/sole parent family/shared care/other	99/613	16.1	2.50	1.90–3.29	**< 0.001**
**Current dwelling ^1^**					
Owned/being purchased	186/2314	8.0	1.00		
Rented from government/rented privately/other	58/317	18.4	2.59	1.88–3.57	**< 0.001**
**Highest education of parents ^1^**					
University degree or higher	53/687	7.7	1.00		
Trade/certificate/diploma or lower	191/1948	9.8	1.31	0.95–1.80	0.099
**Long term illness or ongoing pain that puts a strain on family ^1^**					
No	161/2394	6.7	1.00		
Yes	81/236	34.3	7.25	5.31–9.91	**< 0.001**
**Ever unhappy at school ^1^**					
Never/Rarely	62/1778	3.5	1.00		
Sometimes/Often/Always	177/835	21.2	7.43	5.49–10.06	**< 0.001**
**Have a best mate/a group of friends ^1^**					
Yes	178/2500	7.1	1.00		
No	59/127	46.8	11.48	7.85–16.80	**< 0.001**
**Bullied in last month ^1^**					
No	132/2160	6.1	1.00		
Yes (physical/emotional/both)	100/417	24.0	4.85	3.65–6.46	**< 0.001**

^1^ Other/not stated category not reported. ^2^ The “blended family” category within family structure was defined to include grandparents caring for grandchildren and foster care arrangements. Abbreviations: n, number of children with parent-reported mental health problems; N, total number of children in the relevant category; SEIFA, Socio-Economic Index For Areas.

**Table 5.** Multivariable analyses of association between demographic, risk factors and parent-reported mental health problems among children by age group, 2005/06 to 2014/15.

	Children aged 5 to 11 (n = 334/3627)				Children aged 12 to 15 (n = 229/2406)		
	Odds Ratio	95% Confidence Interval	*P* value		Odds Ratio	95% Confidence Interval	*P* value
**Sex**							
Female	–				–		
Male	2.37	1.83–3.08	**< 0.001**		2.95	2.12–4.11	**< 0.001**
**Family structure ^1,2^**							
Living with biological parents	–				–		
Step or blended family/sole parent family/shared care/other	1.52	1.13–2.05	**0.006**		1.65	1.17–2.33	**0.005**
**Current dwelling ^1^**							
Owned/being purchased	–				–		
Rented from government/rented privately/other	1.43	1.03–1.99	**0.033**		1.41	0.94–2.14	0.099
**Long-term illness or pain that puts a strain on family ^1^**							
No	–				–		
Yes	5.04	3.80–6.70	**< 0.001**		4.17	2.84–6.10	**< 0.001**
**Ever unhappy at school^1^**							
Never/rarely	–				–		
Sometimes/often/always	3.45	2.63–4.54	**< 0.001**		5.04	3.58–7.05	**< 0.001**
**Have a best mate/a group of friends ^1^**							
Yes	–				–		
No	3.54	2.47–5.06	**< 0.001**		5.74	3.59–9.18	**< 0.001**
**Bullied in last month ^1^**							
No	–				–		
Yes (physical/emotional/both)	1.58	1.20–2.09	**0.001**		1.91	1.34–2.72	**< 0.001**

^1^ Other/not stated category not reported. ^2^The “blended family” category within family structure was defined to include grandparents caring for grandchildren and foster care arrangements.

**Table 6.** Univariable analysis of association between demographic, risk factors and use of mental health services among children aged 5 to 15 who reportedly had mental health problems, 2005/06 to 2014/15 (n = 292/603).

	n/N	%	Odds Ratio	95% Confident Interval	*P* value
**Age group**					
5 to 11 years	160/357	44.8	1.00		
12 to 15 years	132/243	54.4	1.47	1.06–2.04	**0.021**
**Sex**					
Female	83/186	44.6	1.00		
Male	209/414	50.5	1.27	0.90–1.79	0.182
**Household income ^1^**					
≥ $40,001	177/378	46.8	1.00		
≤ $40,000	78/147	52.8	1.27	0.87–1.86	0.217
**SEIFA quintile ^1^**					
Middle/High/highest quintile	142/323	43.9	1.00		
Low/lowest quintile	149/273	54.6	1.54	1.11–2.12	**0.009**
**Family structure ^1,2^**					
Living with biological parents	177/389	45.5	1.00		
Step or blended family/sole parent family/shared care/other	108/202	53.7	1.39	0.99–1.95	0.060
**Current dwelling ^1^**					
Owned/being purchased	219/461	47.4	1.00		
Rented from government/rented privately/other	73/138	53.1	1.26	0.86–1.84	0.240
**Highest education of parents ^1^**					
University degree or higher	67/136	49.4	1.00		
Trade/certificate/diploma or lower	225/464	48.5	0.96	0.66–1.41	0.850
**Long term illness or ongoing pain that puts a strain on family ^1^**					
No	140/384	36.4	1.00		
Yes	150/214	70.2	4.12	2.87–5.90	**< 0.001**
**Ever unhappy at school ^1^**					
Never/Rarely	59/177	33.1	1.00		
Sometimes/Often/Always	221/407	54.4	2.41	1.67–3.48	**< 0.001**
**Have a best mate/a group of friends ^1^**					
Yes	197/439	44.8	1.00		
No	78/139	56.0	1.57	1.07–2.30	**0.022**
**Bullied in last month ^1^**					
No	130/304	42.9	1.00		
Yes (physical/emotional/both)	143/267	53.7	1.54	1.11–2.15	**0.010**

^1^ Other/not stated category not reported. ^2^ The “blended family” category within family structure was defined to include grandparents caring for grandchildren and foster care arrangements. Abbreviations: n, number of children who had parent-reported mental health problems and used mental health services; N, total number of children with parent-reported mental health problems in the relevant category; SEIFA, Socio-Economic Index For Areas.

**Table 7.** Multivariable analyses of association between demographic, risk factors and use of mental health services among children aged 5 to 15 years who reportedly had mental health problems, 2005/06 to 2014/15 (n = 292/603).

	Odds Ratio	95% Confidence Interval	*P* value
**Age group**			
5 to 11 years	–		
12 to 15 years	1.55	1.09–2.22	0.015
**Sex**			
Female	–		
Male	1.48	1.01–2.17	0.043
**Long term illness or ongoing pain that puts a strain on family ^1^**			
No	–		
Yes	4.10	2.82–5.94	< 0.001
**Ever unhappy at school ^1^**			
Never/Rarely	–		
Sometimes/Often/Always	2.35	1.59–3.49	< 0.001

^1^ Other/not stated category not reported.

## 4. Discussion

The present study suggests that about one in ten SA school-aged children had parent-reported mental health problems. The figure is comparable to an earlier Australian survey [26] and considerably similar to that reported in the most recent national survey [8], when mental health abnormalities were assessed using the SDQ based on parent or carer reported information (9.9% for children aged 4–11 years and 10.3% for children aged 12 to 16 years). SDQ is a validated instrument that has been widely used in many large children mental health studies in Australia [13]. Unlike other studies, our study used a single-question SDQ item to define the mental health difficulties in children and therefore, was unable to provide comprehensive information on the specific domains of any mental health problems. However, the demonstrated capability of this single-item approach [21] can be almost as good as using a full scale questionnaire for the purpose of monitoring prevalence of overall mental health problems, informing policy making and early intervention planning. Discrepancies between parents' and adolescents' reports of perceived mental health impairment have been previously reported [8], showing much higher prevalence if adolescents themselves provided the information. However, parents' report of mental health problems is relevant to young children and those in their early-adolescence as in the current study, providing important perspective to the issue.

Results of the present study indicate that the prevalence of parent-reported mental health problems among SA school-age children have remained unchanged over the past decade. Costello et al [27] reviewed over twenty studies and suggested that the prevalence of child or adolescent depression had not increased in the past 30 years using clinical diagnostic criteria of mental health problems. The 2013–14 Australian Child and Adolescent Survey of Mental Health and Wellbeing [8] also did not reveal differences in the prevalence of overall mental health problems among children and adolescence by comparing the data collected in 1998 and those collected in 2013.

Consistent with previous findings, the prevalence of parent-reported mental health problems was higher among boys compared to girls in our study. This may be explained partially by the fact that girls have more depressive disorders that may not be noticed by their parents, while boys have more behavioural problems which could be easily identified [8]. Also, girls were more likely to demonstrate a higher level of mental health competence as compared to boys [17].

Results from this study suggest that SA school-aged children who suffered from chronic illness or pain were more likely to have parent-reported mental health problems. It has been reported that psychological well-being and health-related quality of life was poor among young adults with childhood-onset chronic physical conditions [28]. A US study showed that mental health status and prevalence of anxiety may remain stable over time in childhood cancer survivors [29]; however, this may depend on whether a physical function disabling condition had developed during the course of the disease [30]. Alternatively, the increased level of psychosomatic complaint could either be a consequence or symptom of mental health problem experienced by the child.

Our study shows that adverse school experiences increase the likelihood of having mental health problems among children. School experiences may be an important determinant of mental health problems in children between 9 to 12 years [31]. Children who had been involved in school bullying events since primary school were at greater risk of developing mental health problems at 18 years of age, according to a recent longitudinal population-based study [32].

The present study shows that not living with biological parents is associated with increased risk of mental health problems. A similar link has been evident in earlier studies [33], which may relate to poor attachment to, or poor functioning of, a family [34]. For school-aged children, family remains an important domain of their social life. It has been reported that frequent contact with biological parents benefits the mental health of children in foster care [35] while mental health complaints substantially increase in children after loss of contact with their fathers following divorce as compared to those who had preserved parental contact [36].

The present study indicates that living in a rental property is associated with increased risk mental health problems among the younger children. Living in a rental dwelling may represent a low or unstable household income. Mental health problems are more common in individuals with lack of financial and social means [15],[37]. It has been suggested that targeting individual resilience is not enough to offset the effects of the social and environmental contexts such as economic adversity and limited social connections [38]. Thus it is important to refine health promotion messages and support services to cater for these demographic groups using a population approach.

Comparable to the findings of the second Australian Child and Adolescent Survey of Mental Health and Wellbeing survey [8], results of the current study show that around half of SA children with mental health problems had ever received professional help; which is higher than the 29% reported in the first survey [12]. This can perhaps be explained by restriction by the authors to treatment within the last six months in the first survey as opposed to having ever been treated which is the time framed used in our study. Regardless, it nonetheless supports the notion that large numbers of children are potentially missing out when it comes to receiving support. Results of the present study fail to reveal any possible barriers to mental health care among SA children. A New South Wales survey of parents suggested that financial strain and accessibility of mental health services are important barriers to care [39]. Parental attitude towards diagnosis and family dysfunction also serve as barriers to mental health care of children and adolescents [40]. Young people are often reluctant to seek professional help for their mental health problems. Help-seeking barriers among young people are related to their beliefs of self-reliance, lack of information and perceived stigma attached to mental health problems [41],[42].

An interesting finding of our study is that the proportion of school-aged children who had ever been treated for their mental health problems increased over time during the past decade. Increased use of mental health services without substantial increase in mental health problems among Finnish-speaking children over two decades has also reported in a recent study [43]. Traditionally, parents are more likely to seek informal help than professional help [44]. However, increase in service utilisation is not unexpected since the increasing interests in mental health and awareness of mental health services among the public brought by campaigns in recent years. Mental health services among older children and adolescents who experience mental health problems might have increased, given that online mental health services are readily available [45]; however, this cannot not be detected based on parent-reported information as in the present study.

Although it has been suggested that boys are more negative in their attitudes towards mental health education [46], our findings show that boys were more likely than girls to have ever been treated for their mental health problems. Seeking professional help among school-aged children is still heavily influenced by their parents and their perception of problems [41],[47]. Our study shows that older children are more likely to have received mental health care than younger children. This is in agreement with the findings of a longitudinal study, which demonstrated an increase in overall rates of mental health care use as the children growing older from 10 years old to 19 years old [48]. This higher life-time-prevalence of mental health service use among older children may be explained that they were older and had had more time to develop problems and receive treatment as compared to the younger ones.

Earlier research using SAMSS data [49] has reported that school-aged children who had asthma and those who felt unhappy at school were also more likely to be treated with mental health problems. This is substantiated by the findings of the current study. Kuo et al. [50] found that the behavioural and mental health concerns were the major reasons for health care visits among children who had complex medical needs, suggesting an integrated approach in health care is needed for children with chronic conditions.

School experiences play a significant role in the development of childhood mental health problems. Findings of the present study support the need of school mental health services and school-based intervention. Making use of school services provides access and may circumvent possible costs involved with pursuing other specialised professional services; however, it is not without challenge in terms of resources [51],[52]. In addition, school-based promotion and intervention may be more beneficial for children who may not necessarily meet criteria for a clinical diagnosis but have a higher risk of missing out on specialised treatment. Schools have been the largest providers of mental health care to children and adolescents in the US [53]. Tailoring mental health education for the school environment, involving educators, parents and children, as well as improving training and support therefore becomes an increasingly warranted strategy [54],[55].

A major strength of the present study is using the same criteria so that trend or changes over time can be monitored, providing useful information to assist a timely response (early identification and intervention) to changes in prevalence. The second strength of this study is the use of a large representative population-based sample, allowing for more reliable generalisation of the results. We recognise that there are also limitations of this study. Firstly, the use of telephone surveys sourced from the Australian EWP inherently entails the exclusion of population subgroups, namely those with silent numbers and mobile telephone only households. However, the EWP has previously been demonstrated to be an effective tool in monitoring self-report health estimates [56]. Response rates for all telephone surveys have been declining due to increasing awareness of protecting privacy and marketing telephone calls overwhelming potential participants [57]. The response rate of this study can be considered as acceptable and the data has been undertaken using survey weight to minimise the non-response bias [58]. Secondly, as routinely collected data were not designed to answer the research questions specified in the current study, some risk factors of childhood mental health problems, such as parental mental health status, parenting practices and critical life events (loss of close people and experience of violence), were not measured; therefore, not included in the analyses. Finally, using cross-sectional data precludes the ability to establish a direction of causation.

## 5. Conclusion

This study reports population-based prevalence estimates and trends over time of parent-reported mental health problems, as well as health care services utilization for mental health problems among SA school-aged children. Identified mental health problem related risk factors provide useful information for planning of catered population initiatives. Half of the school-aged children with mental health problems did not receive professional treatment, which warrants targeted early interventions.

## References

[1] Waters E, Davis E, Nicolas C (2008). The impact of childhood conditions and concurrent morbidities on child health and well-being. Child Care Health Dev.

[2] Klassen AF, Miller A, Fine S (2004). Health-related quality of life in children and adolescents who have a diagnosis of attention-deficit/hyperactivity disorder. Pediatrics.

[3] Costello EJ, Foley DL, Angold A (2006). 10-year research update review: the epidemiology of child and adolescent psychiatric disorders: II. Developmental epidemiology. J Am Acad Child Adolesc Psychiatry.

[4] Hale DR, Bevilacqua L, Viner RM (2015). Adolescent Health and Adult Education and Employment: A Systematic Review. Pediatrics.

[5] Usami M (2016). Functional consequences of attention-deficit hyperactivity disorder on children and their families. Psychiatry Clin Neurosci.

[6] Houtrow AJ, Larson K, Olson LM (2014). Changing trends of childhood disability, 2001–2011. Pediatrics.

[7] World Health Organisation (2001). The World Health Reprot: Mental Health: New Understanding, New Hope.

[8] Lawrence D, Johnson S, Hafekost J (2015). The Mental Health of Children and Adolescents. Report on the second Australian Child and Adolescent Survey of Mental Health and Wellbeing.

[9] Jorn A, Malhi G (2013). Evidence-based mental health services reform in Australia: where to next. Aust N Z J Psychiatry.

[10] Kessler RC, Amminger GP, Aguilar-Gaxiola S (2007). Age of onset of mental disorders: a review of recent literature. Curr Opin Psychiatry.

[11] An der Heiden W, Hafner H (2000). The epidemiology of onset and course of schizophrenia. Eur Arch Psychiatry Clin Neurosci.

[12] Sawyer MG, Arney FM, Baghurst PA (2001). The mental health of young people in Australia: key findings from the child and adolescent component of the national survey of mental health and well-being. Aust N Z J Psychiatry.

[13] Bayer J, Ukoumunne O, Lucas N (2011). Risk factors for childhood mental health symptoms: National Longitudinal Study of Australian Children. Pediatrics.

[14] Barrett A, Turner R (2005). Family structure and mental health: the mediating effects of socioeconomic status, family process, and social stress. J Helath Soc Behav.

[15] Davis E, Sawyer MG, Lo SK (2010). Socioeconomic risk factors for mental health problems in 4-5-year-old children: Australian population study. Acad Pediatr.

[16] Fox RA, Platz DL, Bentley KS (1995). Maternal factors related to parenting practices, developmental expectations, and perceptions of child behavior problems. J Genet Psychol.

[17] Goldfeld S, Kvalsvig A, Incledon E (2014). Predictors of mental health competence in a population cohort of Australian children. J Epidemiol Community Health.

[18] Lin JD, Hsieh YH, Lin FG (2013). Modification effects of family economic status and school factors on depression risk of single-father family children in Mid-Taiwan area. Res Dev Disabil.

[19] Shin KM, Cho SM, Shin YM (2016). Effects of Early Childhood Peer Relationships on Adolescent Mental Health: A 6- to 8-Year Follow-Up Study in South Korea. Psychiatry Investig.

[20] Taylor A, Dal Grande E (2008). Chronic disease and risk factor surveillance using the SA Monitoring and Surveillance System. (SAMSS) — history, results and future challenges. Public Health Bull South Aust.

[21] Goodman R (1999). The extended version of the Strengths and Difficulties Questionnaire as a guide to child psychiatric caseness and consequent burden. J Child Psychol Psychiatry.

[22] Australian Bureau of Statistics (2013). 2033.0.55.001 Technical Paper: Socioeconomic Indexes for Areas (SEIFA) 2011.

[23] IBM Statistics (NY USA) Statistical Package for the Social Sciences (SPSS), 21.0 for Windows.

[24] StataCorp (College Station, Taxas, US) STATA, version 13.0.

[25] Hosmer D, Lemeshow S (2000). Applied Logistic Regression.

[26] Australian Institute of Health and Welfare (2009). A picture of Australia' children 2009, Part II How healthy are Australia's children?.

[27] Costello E, Erkanli A, Angold A (2006). Is there an epidemic of child or adolescent depression?. J Child Psychol Psychiatry.

[28] Verhoof E, Maurice-Stam H, Heymans H (2013). Health-related quality of life, anxiety and depression in young adults with disability benefits due to childhood-onset somatic conditions. Child Adolesc Psychiatry Ment Health.

[29] Phillips SM, Padgett LS, Leisenring WM (2015). Survivors of childhood cancer in the United States: prevalence and burden of morbidity. Cancer Epidemiol Biomarkers Prev.

[30] Maurice-Stam H, Verhoof EJ, Caron HN (2013). Are survivors of childhood cancer with an unfavourable psychosocial developmental trajectory more likely to apply for disability benefits?. Psychooncology.

[31] Waenerlund AK, Stenmark H, Bergstrom E (2016). School experiences may be important determinants of mental health problems in middle childhood—a Swedish longitudinal population-based study. Acta Paediatr.

[32] Lereya ST, Copeland WE, Zammit S (2015). Bully/victims: a longitudinal, population-based cohort study of their mental health. Eur Child Adolesc Psychiatry.

[33] Meltzer H, Gatward R, Goodman R (2003). Mental health of children and adolescents in Great Britain. Int Rev Psychiatry.

[34] Jozefiak T, Wallander JL (2015). Perceived family functioning, adolescent psychopathology and quality of life in the general population: a 6-month follow-up study. Qual Life Res.

[35] McWey LM, Acock A, Porter B (2010). The Impact of Continued Contact with Biological Parents upon the Mental Health of Children in Foster Care. Child Youth Serv Rev.

[36] Reiter SF, Hjorleifsson S, Breidablik HJ (2013). Impact of divorce and loss of parental contact on health complaints among adolescents. J Public Health (Oxf).

[37] Poole-Di Salvo E, Silver EJ, Stein RE (2015). Household Food Insecurity and Mental Health Problems Among Adolescents: What Do Parents Report?. Acad Pediatr.

[38] Sameroff AJ, Rosenblum KL (2006). Psychosocial constraints on the development of resilience. Ann N Y Acad Sci.

[39] Iskra W, Deane FP, Wahlin T (2015). Parental perceptions of barriers to mental health services for young people. Early Interv Psychiatry.

[40] Radovic A, Reynolds K, McCauley HL (2015). Parents' role in adolescent depression care: primary care provider perspectives. J Pediatr.

[41] Rickwood DJ, Deane FP, Wilson CJ (2007). When and how do young people seek professional help for mental health problems?. Med J Aust.

[42] Reavley NJ, Yap MB, Wright A (2011). Actions taken by young people to deal with mental disorders: findings from an Australian national survey of youth. Early Interv Psychiatry.

[43] Sourander A, Lempinen L, Brunstein Klomek A (2016). Changes in Mental Health, Bullying Behavior, and Service Use Among Eight-Year-Old Children Over 24 Years. J Am Acad Child Adolesc Psychiatry.

[44] Girio-Herrera E, Owens JS, Langberg JM (2013). Perceived barriers to help-seeking among parents of at-risk kindergarteners in rural communities. J Clin Child Adolesc Psychol.

[45] Burns J, Birrell E (2014). Enhancing early engagement with mental health services by young people. Psychol Res Behav Manag.

[46] Williams B, Pow J (2007). Gender differnces and mental health: An exploratory study of knowledge and attitides to mental health among Scottish teenagers. Child Adolesc Mental Health.

[47] Rickwood D, Mazzer K, Telford N (2015). Social influences on seeking help from mental health services, in-person and online, during adolescence and young adulthood. BMC Psychiatry.

[48] Reijneveld SA, Wiegersma PA, Ormel J (2014). Adolescents' use of care for behavioral and emotional problems: types, trends, and determinants. PLoS One.

[49] Collins JE, Gill TK, Chittleborough CR (2008). Mental, emotional, and social problems among school children with asthma. J Asthma.

[50] Kuo DZ, Melguizo-Castro M, Goudie A (2015). Variation in child health care utilization by medical complexity. Matern Child Health J.

[51] Atkinson C, Squires G, Bragg J (2014). Facilitators and barriers to the provision of therapeutic interventions by school psychologists. Sch Psychol Int.

[52] Eiraldi R, Wolk CB, Locke J (2015). Clearing Hurdles: The Challenges of Implementation of Mental Health Evidence-Based Practices in Under-resourced Schools. Adv Sch Ment Health Promot.

[53] Merikangas KR, He JP, Burstein M (2011). Service utilization for lifetime mental disorders in U.S. adolescents: results of the National Comorbidity Survey-Adolescent Supplement (NCS-A). J Am Acad Child Adolesc Psychiatry.

[54] Allen K, McKenzie V (2015). Adolescent mental health in an Australian context and future intervention. Int J Ment Health.

[55] Bayer J, Hiscock H, Scalzo K (2009). Systematic review of preventive interventions for children's mental health: what would work in Australian contexts?. Aust N Z J Psychiatry.

[56] Dal Grande E, Taylor A, Wilson D (2005). Is there a difference in health estimates between people with listed and unlisted telephone numbers?. Aus N Z J Public Health.

[57] Brick J, Williams D (2013). Explaining rising nonresponse rates in cross-sectional surveys. Ann Am Acad Political Soc Sci.

[58] Brick JM (2013). Unit Nonresponse and Weighting Adjustments: A Critical Review. J Off Stat.

